# Does tourism affect the long term course of COVID-19 pandemic in a country of destination? Evidence from a popular Greek island in 2020 where control measures were implemented

**DOI:** 10.3389/fepid.2023.1149706

**Published:** 2023-06-28

**Authors:** Zacharoula Bogogiannidou, Michalis Koureas, Varvara A. Mouchtouri, Katerina Dadouli, Maria A. Kyritsi, Alexandros Vontas, Lemonia Anagnostopoulos, Paraskevi Mina, Alexia Matziri, Evangelia Vachtsioli, Alexandra Papagiannakis, Zacharias Archontakis, Michael Leotsinidis, Kalliopi Theodoridou, George Manios, Achilleas Gikas, Matthaios Speletas, Christos Hadjichristodoulou

**Affiliations:** ^1^ Laboratory of Hygiene and Epidemiology, Faculty of Medicine, University of Thessaly, Larissa, Greece; ^2^ Biochemical Laboratory, Venizeleio Pananio General Hospital, Heraklion, Greece; ^3^ Microbiological Laboratory “Diagnosis Rethimnou”, Rethimno, Greece; ^4^ Laboratory of Public Health, School of Medicine, University of Patras, Patras, Greece; ^5^ Department of Microbiology, Andreas Sygros Hospital, National and Kapodistrian University of Athens, Athens, Greece; ^6^ Department of Computer Science and Biomedical Informatics, University of Thessaly, Lamia, Greece; ^7^ Internal Medicine Department, Infectious Diseases Unit, University Hospital of Heraklion, School of Medicine, University of Crete, Heraklion, Greece; ^8^ Department of Immunology and Histocompatibility, Faculty of Medicine, University of Thessaly, Larissa, Greece

**Keywords:** COVID-19, SARS-CoV-2, seroprevalence, Crete, tourism, long-term impacts, course of the pandemic, destination region

## Abstract

Greece opened its points of entry on July 1, 2020, with specific guidelines for travellers arriving by sea, air or land. The aim of this article is to examine the effect of tourism on the long term course of the Coronavirus Disease 2019 (COVID-19) pandemic during the pre-vaccination era (June to December 2020) on the popular Greek island of Crete. To achieve this, a cross-sectional serosurvey, repeated at monthly intervals, was conducted to compare the seroprevalence in Crete with seroprevalence in the mainland of Greece. Crete welcomed nearly 2,000,000 travellers during the 2020 summer season. Left-over serum samples were collected and obtained from public and private laboratories located in Greece, including the island of Crete. These samples were tested for the presence of anti-SARS-CoV-2 IgG antibodies. A total of 55,938 samples were collected, 3,785 of which originated from Crete. In Crete, the seroprevalence ranged between 0% (June 2020) and 2.58% (December 2020), while the corresponding seroprevalence in Greece was 0.19% and 10.75%, respectively. We identified 4.16 times lower seropositivity in Crete (2.58%) in comparison with the mainland of Greece (10.75%) during December 2020. Moreover, the monthly infection fatality rate (IFR) in Crete was calculated at 0.09%, compared with 0.21% in mainland Greece for December 2020. The island of Crete presented more than four times lower seroprevalence than the mainland of Greece, despite being a highly attractive tourist destination. This evidence supports the idea that tourism may not have affected the long term course of the COVID-19 pandemic in Greece. However, due to contradicting results from previous studies, further investigation is needed.

## 1. Introduction

### 1.1. Reopening tourism via travel-related public health measures

On 1 July 2020 the entry of tourists into Greece was permitted through 41 points of entry, with 27 airports, 7 ports and 7 ground crossings open to international arrivals. The completion of a Passenger Locator Form (PLF) was required at minimum 48 h prior to check in/arrival for all tourists arriving by air, sea and land. Within this form, detailed information was collected regarding the travellers’ point of departure, duration of previous stays in other countries and address of their stay in Greece. Targeted testing upon arrival via polymerase chain reaction (PCR) was performed at Greek points of entry (1). The epidemiology of Coronavirus Disease 2019 (COVID-19) in the country of origin or in countries previously visited before travelling to Greece, were among the risk assessment criteria used in a testing algorithm upon arrival. After completion of the PLF a personal quick response (QR) code was sent to the traveller and based on the information contained therein, travellers were classified into one of two categories. Depending on their unique QR code, screening personnel either directed travellers to a screening area or allowed them to enter the country. Travellers tested for severe acute respiratory syndrome coronavirus 2 (SARS-CoV-2) by a trained health team were recommended to self-isolate at the address of their final destination (as declared in their PLF) until screening results were available, with quarantine ending provided that the test result was negative.

Reopening of borders during the summer of 2020 reflected one stage of lifting restrictive measures implemented in the context of COVID-19. A national lockdown beginning on 23 March 2020 was imposed in an effort to reduce the spread of SARS-CoV-2, by which point 695 COVID-19 cases had been recorded in Greece with only five in Crete; four COVID-19 cases had been recorded in the regional unit (RU) of Heraklion and one case in the RU of Lasithi. Beginning 4 May 2020, the national lockdown was gradually lifted with a requirement for face masks to be worn in areas of intense crowding (including means of public transport, supermarkets, hospitals etc.). A total of 2,632 cases were recorded up to the start of lifting restrictive measures, and all RUs of Crete (Heraklion, Lasithi, Chania and Rethimno) presented a low COVID-19 incidence with 0–4 cases per 100,000 population. On 18 May 2020, mobility between RUs within the country was permitted and in late May 2020, eating establishments were reopened. Between mid-May to June 2020, schools gradually reopened with senior classes the first to commence. From 15 June 2020 the two busiest Greek airports located in Athens and Thessaloniki reopened, accepting flights from specific countries; starting 1 July 2020 the entry of tourists into Greece via any airport from all countries was permitted.

Crete has two national airports located in Heraklion and Chania, as well as a municipal/local airport located in Lasithi RU. Ports are located in each of Crete’s four RUs. From January to June 2020 (which includes the period of national lockdown), a total of 161,736 and 35,304 arrivals were recorded by air to Heraklion and Chania, respectively. After reopening the points of entry in Greece, a total of 198,868 arrivals by air were recorded in Heraklion and 46,693 in Chania in July, followed by: 249,104 and 61,101 in August; 183,556 and 41,727 in September; 103,619 and 16,127 in October; 5,393 and 829 in November for Heraklion and Chania, respectively. Moreover, during the summer period nearly 1,200,000 tourists arrived on ferries through Crete’s ports.

### 1.2. The aim of our study

The aim of this study was to consider if the presence of a high level of tourism and interaction of tourists is sufficient to affect the long term course of COVID-19 in the destination region. We assessed the seropositivity of the population of Crete—a tourist-based island—compared to the entire population of Greece, in order to investigate if there is any possible effect of the tourist wave on the long term course of COVID-19 pandemic.

## 2. Materials and methods

### 2.1. Study design and participants

The study was initially designed as a national cross-sectional survey—including Crete—and repeated at monthly intervals (2–4). We used the leftover sampling methodology in order to collect serum samples (residual sera) from the general population throughout Greece. As we described in already published scientific articles, a geographically stratified sampling plan based on regional units [Nomenclature of Territorial Units for Statistics (NUTS) level 3] was applied in order to produce a representative sample, taking into consideration age group (0–29, 30–49, 50–69, and ≥70 years) and sex (2–4). The required sample size was determined to be 380 serum samples from each of the 13 NUTS level 2 regions and the sample size for each regional unit (NUTS level 3) from the corresponding region was calculated according to population distribution. As a result, 380 serum samples corresponded to Crete. However, the actual number of collected samples differed from the pre-determined number of samples above. As described in more detail below, we adjusted our results according to the regional unit’s population and their characteristics, sex and age.

The leftover serum samples were collected from a nationwide laboratory framework, including both private microbiological laboratories as well as microbiological and biochemical laboratories of public hospitals in Greece. During the study period, six of the participating laboratories were located in Crete. The samples were derived from individuals who visited the laboratories for routine screening and reasons unrelated to COVID-19. All the confirmed COVID-19 cases were excluded from the sampling. Here, we present the results for the region of Crete, compared to the entire country from June to December 2020, as Crete is a popular tourist-based island that attracts a high influx of tourists.

We have already published findings regarding SARS-CoV-2 seroprevalence in Greece during the pre-vaccination pandemic era, from March to December 2020 (2–4). According to our results, a higher seroprevalence was calculated in December 2020 among younger age groups of “0–29” and “30–49”, as well as in highly populated metropolitan areas (4).

### 2.2. Storage and shipment of the leftover samples

All the participants’ blood samples were collected in a sterile tube. Whole blood was allowed to clot and then centrifuged to separate the serum. The serum was carefully removed with a fine-bore pipette to avoid extracting red cells, and transferred aseptically to a sterile vial labelled with the patient’s identifier, date of collection and specimen type. The leftover serum samples were frozen at −20°C or lower and transported to our laboratory on frozen ice packs appropriately and as soon as possible. They were placed sealable in plastic bags containing absorbent materials. Styrofoam boxes were used to contain the sealed bags. The frozen ice packs were placed at the bottom and along the sides of the styrofoam box. The samples were placed in the centre and more ice packs were placed on top. After receiving of the samples, we tried to test them as soon as possible. In case this was not possible, they were stored in −80°C.

The same method of samples’ preservation and shipment was followed either in the case of private or public laboratories. All the required materials for samples storage and transport were sent from our laboratory in order to the appropriate conditions to be ensured.

### 2.3. Laboratory analysis

As we mentioned in previous articles, the presence of anti-SARS-CoV-2 IgG antibodies was determined using the AB-BOTT SARS-CoV-2 IgG assay, a chemiluminescent microparticle immunoassay (CMIA), with the ARCHITECT i2000SR analyzer (Abbott, Illinois, United States) (2–4). Anti-spike IgG antibodies are used as a marker of prior SARS-CoV-2 infection. The method was validated in our laboratory. We used 305 pre-COVID-19 samples (obtained in 2017) as negative controls and 94 samples from patients with positive SARS-CoV-2 PCR and different symptom durations. The kit displayed 84.0% sensitivity [95% confidence interval (CI): 76.6–91.5] and 99.7% specificity (95% CI: 98.2–100). Given that vaccines were not available during the study period (June–December 2020), all positive samples for IgG anti-SARS-CoV-2 were provoked by natural infection.

### 2.4. Statistical analysis

The statistical analysis applied is identical to the analysis applied for samples between March to April 2020 (2) and May to August 2020 (3).

#### 2.4.1. Weighted prevalence

Initially, we determined an unweighted relative frequency of all patient characteristics (age, sex and area of residence): this is the crude seroprevalence (S1). The weighted proportions of positive tests in the countrywide sample were based on the sex and age distribution within each regional unit (NUTS level 3) and the population of each regional unit, according to the most recent census conducted in 2011 (S2) (5). We also adjusted the weighted proportion (S2) of positive tests to account for the accuracy (sensitivity and specificity) of the laboratory test (S3) (6, 7). Since reported COVID-19 cases were by definition outside the sampling framework, the seroprevalence was corrected taking into consideration the number of reported cases per month, in accordance with the National Public Health Organization (NPHO) (S4). Therefore, we added the cases reported in March, April, May and June to the estimated S3 seroprevalence in order to calculate the S4 for June, while to calculate the S4 for July we added the reported cases from March to July and so forth. We calculated the S1, S2, S3 and S4 seroprevalence of IgG antibodies by month, in addition to calculating the case fatality rate (CFR) and infection fatality rate (IFR) by month. The CFR is the ratio of the number of deaths attributed to COVID-19 and reported to the NPHO, divided by the number of cases reported to the NPHO; the IFR is the ratio of deaths divided by the number of estimated individuals infected with SARS-CoV-2. The estimation of infected individuals was the product of the seroprevalence and population of regional units, where confirmed cases were identified according to NPHO (8). The 95% CI for weighted data were estimated using normal approximation of binomial distribution and effective sample size, rather than the collected sample size (further explained below). The 95% CI for CFR was calculated using normal approximation of binomial distribution. The 95% CI for IFR was calculated using the corresponding 95% CI of the S1, S2, S3 and S4 seroprevalence, with the methodology described in our previous published article (4). Comparison of two proportions was carried out with the ‘*N*−1’ *χ*^2^ test (8).

#### 2.4.2. Effective sample size

Since the number of collected samples from each regional unit was not proportional to the regional unit’s population, we calculated an effective sample size based on each regional unit’s population proportion, according to 2011 census data. This was done using target weighting. The target sample size for a regional unit *i* is *t_i_*, and the actual sample size for the regional unit *i* is *a_i_*. The weighting factor for the regional unit *i* is calculated with the following formula:

(1) $$f_{i}=\frac{t_{i}}{a_{i}}$$

The weighted sample size (*w_i_*) for the regional unit *i* is calculated as follows:

(2) $$w_{i}=t_{i}\times f_{i}.$$

For *k* regional units and a countrywide target sample size of *n_t_*, the country-wide effective sample size (*n_e_*) is calculated with the following formula:

(3) $$n_{e}=\frac{n_{t}^{2}}{\sum_{i=1}^{k}w_{i}}.$$

This can also be written as:

(4) $$n_{e}=\frac{{\left(\sum {\;}_{i=1}^{k}t_{i}\right)}^{2}}{\sum {\;}_{i=1}^{k}(t_{i}^{2}/a_{i})}.$$

### 2.5. Ethical statement

The samples were anonymized leftover serum samples. Each sample had a unique code and the required data—sex, age, residence and date of blood sampling—were recorded. Health staff from the participating laboratories explained that in the context of a cross-sectional serosurvey, there was possibility of antibodies testing against SARS-CoV-2 and requested consent of the participants. The research protocol was approved by the ethical committee of the Faculty of Medicine, University of Thessaly, Greece (No. 2116).

## 3. Results

### 3.1. Seroprevalence per month

Of the total amount of collected samples (55,938 samples), 3,785 samples originated from Crete for the seven-month period between June and December 2020 and 2,170 of them (57.33%) were obtained from females. For each sample, age, sex, and date of blood sampling were recorded.

Of the 3,785 collected serum samples, 39 (1.03%) were found positive for anti-SARS-CoV-2 IgG antibodies. According to the monthly distribution of samples, S1 seroprevalence for anti-SARS-CoV-2 IgG antibodies was as follows: 0.23% in June, 0.53% in July, 0.32% in August, 0.72% in September, 0.96% in October, 1.89% in November and 2.41% in December 2020 (Supplementary Table S1: Anti-SARS-CoV-2 IgG antibody seroprevalence, Crete, June 2020, Supplementary Table S2: Anti-SARS-CoV-2 IgG antibody seroprevalence, Crete, July 2020, Supplementary Table S3: Anti-SARS-CoV-2 IgG antibody seroprevalence, Crete, August 2020, Supplementary Table S4: Anti-SARS-CoV-2 IgG antibody seroprevalence, Crete, September 2020, Supplementary Table S5: Anti-SARS-CoV-2 IgG antibody seroprevalence, Crete, October 2020, Supplementary Table S6: Anti-SARS-CoV-2 IgG antibody seroprevalence, Crete, November 2020, Supplementary Table S7: Anti-SARS-CoV-2 IgG antibody seroprevalence, Crete, December 2020). The adjusted results for age, sex, population (S2) and additionally, for accuracy of the laboratory test (S3) are presented in Supplementary Tables S1–S7. After the addition of NPHO data, S3 seroprevalence was modified and S4 was calculated as 0% in June, 0.09% in July, 0.03% in August, 1.22% in September, 1.41% in October, 1.45% in November and 2.58% in December 2020 (Supplementary Tables S1–S7). A percentage <1% for S4 is observed throughout the summer months, while from September onwards a continuous increase is calculated.

### 3.2. Seroprevalence per age group

**Figure 1:**
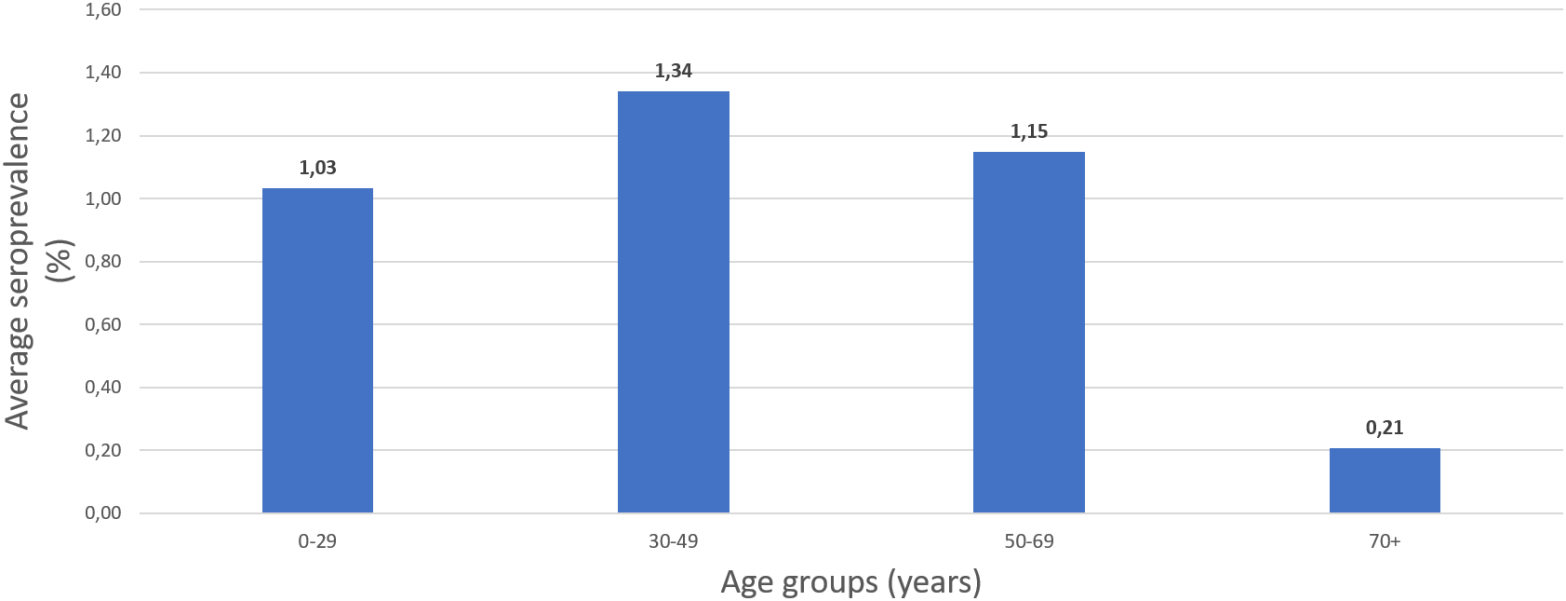
The average seroprevalence of IgG anti-SARS-CoV-2 antibodies per age group in Crete, June–December 2020.

Throughout the study period, the most affected age group varied from month to month. For the first three months between June to August, imperceptible differences existed between age groups. However, from September onwards the difference between age groups increased. In September, the “50–69” presented as the most affected age group with S4 = 4.87% while the next two months the two youngest age groups presented higher seroprevalence (October, “0–29”: S4 = 2.63%, “30–49”: S4 = 1,97%, November: “0–29”: S4 = 1.24%, “30–49”: S4 = 3.32%). During December, the seroprevalence in each age group ranged from S4 = 2.13% to S4 = 3.62%, with the exception of the oldest age group whose seroprevalence was calculated as S4 = 0.94%. To better estimate which age group was more affected, we calculated the average seroprevalence for this seven-month period per age group (Figure 1), which proves that the “30–49” age group was the most affected (S4 = 1.34%).

### 3.3. Seroprevalence per sex

Although males generally presented higher seroprevalence during the study period, no statistically significant difference was observed among the two sexes, apart from the month of October; the S4 was 2.41% in males and 0.42% in females (difference = 4.11%, *p* = 0.015) (Supplementary Table S5).

### 3.4. Comparison of seroprevalence in Crete with the rest of Greece

**Figure 2:**
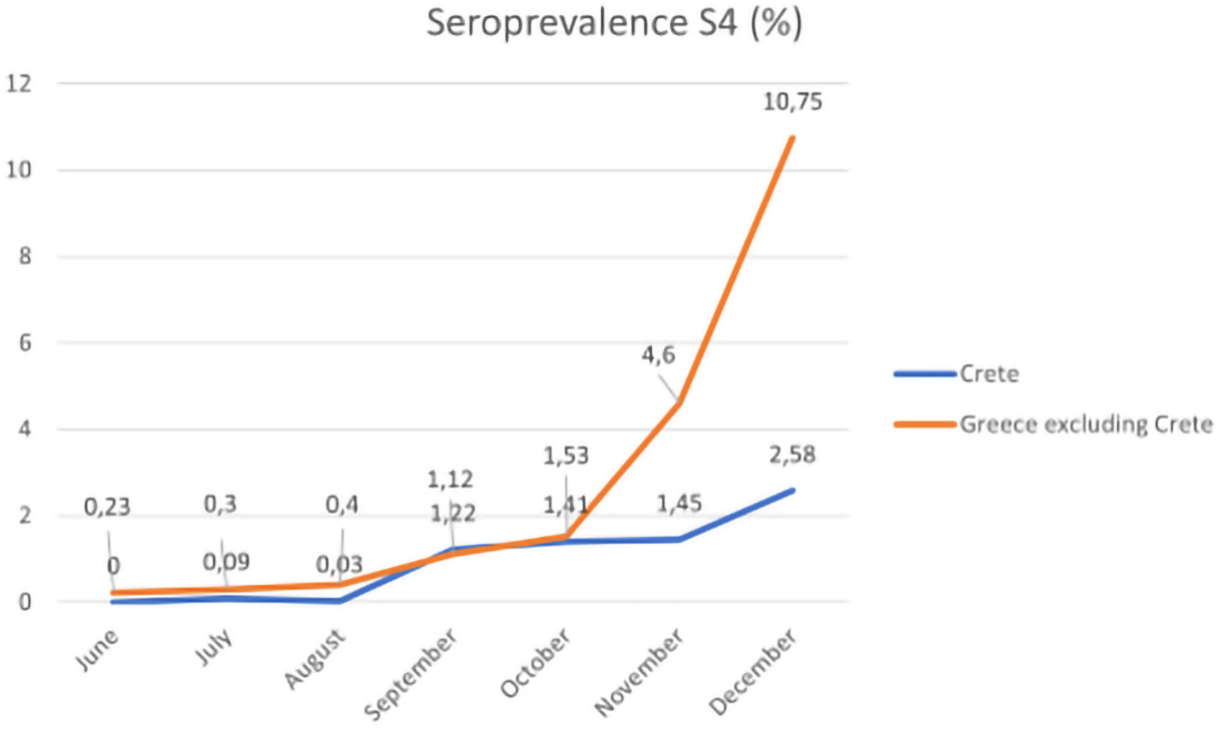
The monthly seroprevalence of IgG anti-SARS-CoV-2 antibodies in Crete and mainland of Greece, June–December 2020.

Using our data derived from the serosurvey conducted for the whole of Greece, we designed the following diagram which presents the S4 in Greece (excluding Crete) compared to the S4 in Crete during the study period (Figure 2). A trend of increasing seroprevalence is observed beginning in September for both the mainland of Greece and Crete; however, the seropositivity is 4.16 times higher in Greece than in Crete (10.75% vs. 2.58%).

### 3.5. S4 seroprevalence, CFR and IFR per month

The monthly CFR and IFR are presented in Supplementary Tables S1–S7. Both values remained low and <1% for each of the first four months (June–September). From October onwards, a continuous increase in CFR is observed, which varying between 1.29% and 7.77%. However, IFR remains low and ranges between 0.00% and 0.10%. In Table 1, the monthly S4, CFR and IFR of COVID-19 for both Crete and mainland Greece are summarized for the study period, based on the S4 seroprevalence. Information regarding the CFR and IFR for the mainland of Greece was derived from the previously published article about SARS-CoV-2 serosurveillance in Greece during the pre-vaccination pandemic era (2–4).

**Table 1:** The monthly S4 seroprevalence, CFR, and IFR of COVID-19 in Crete and the mainland of Greece, June–December 2020.

			June	July	August	September	October	November	December
Crete	Total		0	0.09	0.03	1.22	1.41	1.45	2.58
	Age groups	0–29	0	0	0.03	0.73	2.63	1.24	2.59
		30–49	0.13	0.13	0.31	0.03	1.97	3.32	3.62
		50–69	0.74	0.74	0.03	4.87	0.04	0.22	2.13
		≥ 70	0.15	0.15	0.02	0	0.02	0.31	0.94
	CFR		0	0	0.49	0.84	1.29	1.37	7.77
	IFR		0	0	0.08	0.01	0.02	0.10	0.09
Mainland of Greece	Total		0.23	0.30	0.40	1.12	1.53	4.60	10.75
	Age groups	0–29	0.02	0.07	0.17	1.07	1.34	3.26	12.98
		30–49	1.10	0.53	0.73	1.22	2.00	5.53	11.09
		50–69	0.05	0.33	0.42	0.54	2.01	5.04	10.10
		≥ 70	0.02	0.18	0.08	1.89	0.09	3.05	5.98
	CFR		4.51	2.03	1.16	1.54	1.03	2.72	7.24
	IFR		0.04	0.03	0.10	0.09	0.09	0.39	0.21

## 4. Discussion

Several recent studies have investigated the impact of the COVID-19 pandemic on tourism (9, 10), while very few consider the opposite to examine the impact of tourism on COVID-19 outbreaks. Both previous literature (11) and more recent COVID-19 related literature (9, 10) demonstrate that infectious disease outbreaks have led to sharp and immediate reductions in tourism among affected countries. However, the reverse association is unclear; does tourism determine the course of a pandemic, affecting it over the long-term for months in the country of destination?

As previously mentioned we considered the example of Crete, a Greek island that attracted approximately 2,108,000 travellers, nearly half of the 3,985,000 tourists arrived in Greece in 2020. Meanwhile, Crete covers only about 6% of the Greek population according to the latest national census data and 6.31% of the total Greek area. Crete had four times lower COVID-19 seroprevalence than the mainland of Greece in December 2020, demonstrating that despite the arrival of tourists during previous months, the long term course of the COVID-19 pandemic on the island was not affected. The interpretation of this finding is not only interesting, but it is also valuable to consider the reasons why this occurred. This finding contributes to and informs competent authorities’ evidence-based decision-making.

### 4.1. Probable interpretations of our results

#### 4.1.1. Travel-related public health measures, tourists’ profile and limited interaction between travellers and local population

One probable interpretation was the effectiveness of travel-related public health measures. As extensively described above, during each stage of a tourists’ journey confirmed and suspected COVID-19 cases were isolated and quarantined, respectively. This prevented further SARS-CoV-2 transmission to local populations and mitigated an increased epidemiological viral load in the destination region. Almost half of tourists arrived by air and the majority of foreign tourists who arrived by air came from Germany, followed by France, UK, Netherlands, Switzerland, Italy and Belgium, countries with low COVID-19 incidence though summer period (12–14). In general, the positivity rate for the PCR conducted at all the points of entry in Greece was between 2/1,000 and 5/1,000 with variations among point of entry and week of testing. Our laboratory during the summer of 2020 covered two points of entry with PCR testing. We examined in total 5,648 samples and we identified 13 positive tourists (2.3/1,000). Usually, the tourists are formulating smaller social groups (only with their family, their friends) avoiding substantial interaction with other local people. Tourists may have interacted with working personnel as waiters but staff were required to wear protective masks and follow personal hygiene and public health measures. Therefore, virus transmission was limited to the bubble of tourists.

#### 4.1.2. The pathogen-stress theory

The limited interaction between travellers and locals may be explained by the pathogen-stress theory. This theory proposes that collectivism, vs. individualism, protects the group from pathogens (15). People prefer more in-group social interaction rather than contact with foreigners and other out-group members. This attitude inhibits exposure to novel pathogens such as SARS-CoV-2. Consequently, both the local population and tourists chose in-group social interaction, contributing to limiting transmission of the virus.

#### 4.1.3. Examples of COVID-19 clusters without long-term impacts

The absence of tourism’s long-term impacts on the course of the pandemic is further supported by the example of COVID-19 clusters-outbreaks, which occurred the following year during Easter and summer holidays. Those clusters were primarily attributed to internal tourism where less stringent public health measures were implemented. That tourist wave was largely comprised of young Greek adults who travelled to islands such as Mykonos and Paros, interacted primarily with their peers and other travellers, and did not interact with local populations. During their stay on these islands a short-term increase in weekly incidence of COVID-19 cases was observed, with no long-term effect or determination of the pandemic course in the local community.

#### 4.1.4. Seasonality

Many studies have investigated the seasonality of COVID-19, with findings claiming temperature may be negatively related to COVID-19 incidence (16, 17). Another factor under investigation which may affect SARS-CoV-2 transmissibility is exposure to ultraviolet (UV) irradiances of natural sunlight (18) Crete which is located on the south Mediterranean Sea, presents usually sunny days with high temperatures during the summer period. These environmental conditions combined with mainly outdoor activities conducted by tourists during the summer months may have reduced SARS-CoV-2 transmission.

#### 4.1.5. Supporting our findings from a phylogenetic point of view

Our findings are also supported by molecular epidemiology studies. A specific study conducted during summer 2020 in which a Bayesian phylogeographic approach was applied, shows that lifting of travel restrictions and the tourist wave were not associated with onward transmission driven by imported SARS-CoV-2 cases (19). Despite lifting travel restrictions, virus importation remained low and did not substantially contribute to SARS-CoV-2 onward transmission, due to efficient targeted public health measures.

#### 4.1.6. Adapting to current conditions

The measures described above refer to a non-vaccination era; however, we must assess the usefulness of these measures in terms of a highly vaccinated population. To date, more than 80% of the Greek population is vaccinated and in some countries from which the tourists are originated the vaccination coverage is even higher. It is known that vaccinated individuals are at risk of infection, but the risk of hospitalization or death is lower compared to unvaccinated individuals (20). Thus, the measures for travellers should be proportional to the risk and testing at the points of entry or quarantine of travellers has no place in the vaccination era.

### 4.2. A subject that requires further study

However, there are several studies supporting the opposing view, that travellers not only affect the running incidence temporally, but also have long-term epidemiologic impacts inducing higher SARS-CoV-2 transmission rates, determining even further the COVID-19 pandemic course in the region/country of destination (21, 22). Consequently, tourism destination attractiveness is presented as a possible indicator of areas that would be most affected by the pandemic. Certainly, further investigation must be conducted in this field considering many factors, including whether public health travel-related measures are implemented, epidemiological status of the destination region and the SARS-CoV-2 variants that are circulating or introduced.

## 5. Limitations

Our study presents some limitations: (1) the leftover sampling methodology could be considered a limitation of the study, as non-random convenient sampling may affect the representativeness of samples collected; (2) sample collection process was also challenging due to summer closures of many microbiological laboratories, primarily in August 2020; (3) not all of Crete’s RUs were sufficiently covered by the sampling framework for the entire study period; (4) a proportion of SARS-CoV-2 infected may have not developed antibodies against SARS-CoV-2 and (5) there are also individuals who lose their immunity. According to CDC, this proportion was calculated in 28.2% after 2 months of SARS-CoV-2 infection (23). We assume that both the fact that some individuals do not develop antibodies or some others are losing their antibodies after a period of time does not affect the comparison between Crete and the mainland of Greece since the same limitations are encountered in both regions. This methodology outweighs limitations due to ease of sample collection which allows for repeated monthly sampling, and enables follow up of the pandemic’s course and general population’s immunity levels on a rolling basis.

## 6. Conclusions

In conclusion, in this serosurvey we found that Crete had four times lower seroprevalence than the mainland of Greece (Crete: 2.58% VS. mainland Greece: 10.75%). This finding, based on the example of Crete as a popular tourist destination, may indicate that tourism did not affect the long term course of the COVID-19 pandemic in Greece. Reopening of borders with the implementation of public health travel-related measures (such as completion of PLFs, target sampling etc.) limited interaction with local populations, attitude based on pathogen-stress theory, high temperatures and UV irradiance exposure may be some of the reasons which reduced the SARS-CoV-2 transmission. However, since other studies found contradicting results further investigation is needed. The clarification of this issue via a continuously growing body of literature, will contribute to more effective management of the COVID-19 pandemic.

## Data Availability

The raw data supporting the conclusions of this article will be made available by the authors, without undue reservation.
