# Supplementary material for: Does tourism affect the long term course of COVID-19 pandemic in a country of destination? Evidence from a popular Greek island in 2020 where control measures were implemented
Source: Front Epidemiol. 2023 Jun 28;3:1149706. doi: 10.3389/fepid.2023.1149706 (PMC10955759; doi:10.3389/fepid.2023.1149706)
Supplement: Supplementary file 1 [file Table1.docx]

Supplementary Material

**Table 1.** Anti-SARS-CoV-2 IgG antibody seroprevalence, Crete, June 2020

| **June** | | **Positive/**  **sample size** | **S1: Crude prevalence** | | **S2: Age, sex and population-adjusted prevalence** | | **S3: S2 + adjustment for sensitivity and specificity** | | **S4: S3 + NPHO ^1^ data  ^*^** | |
| --- | --- | --- | --- | --- | --- | --- | --- | --- | --- | --- |
|  |  | **n/N** | **Prevalence (%)** | **95% CI ^3^** | **Prevalence (%)** | **95% CI** | **Prevalence (%)** | **95% CI** | **Prevalence (%)** | **95% CI** |
| **Total** | | 2/863 | 0.23 | 0-0.55 | 0.07 | 0–0.24 | 0 | 0–0.01 | 0 | 0–0.01 |
| **Age group**  **(years)** | **0–29** | 1/234 | 0.43 | 0-1.26 | 0.09 | 0-0.46 | 0 | 0-0.19 | 0 | 0-0.19 |
|  | **30–49** | 1/259 | 0.39 | 0-1.14 | 0.13 | 0-0.56 | 0 | 0-0.31 | 0 | 0-0.31 |
|  | **50–69** | 0/225 | 0 | | 0 | | 0 | | 0 | |
|  | **≥ 70** | 0/145 | 0 | | 0 | | 0 | | 0 | |
| **Sex** | **Male** | 1/332 | 0.30 | 0-0.89 | 0.08 | 0–0.38 | 0 | 0–0.09 | 0 | 0–0.09 |
|  | **Female** | 1/531 | 0.19 | 0-0.56 | 0.06 | 0–0.27 | 0 | 0–0.01 | 0 | 0–0.01 |
| **‘Ν-1’ chi-squared test**  **Difference between sex** | | | Difference = 0.11%  p = 0.744 | | Difference = 0.02%  p = 0.913 | | NA ^5^ | | NA | |
| **CFR ^2^ (%)** | | **95% CI** | **IFR according to** | | | | | | | |
|  |  |  | **S1** | | **S2** | | **S3** | | **S4** | |
|  |  |  | **IFR ^4^ (%)** | **95% CI** | **IFR (%)** | **95% CI** | **IFR (%)** | **95% CI** | **IFR (%)** | **95% CI** |
| 0 | | | 0 | | 0 | | 0 | | 0 | |

^1^ NPHO: National Public Health Organization; ^2^ CFR: case fatality rate; ^3^ CI: confidence interval; ^4^ IFR: infection fatality rate; ^5^ NA: not applicable *NPHO: 0 cases, 0 deaths

**Table 2.** Anti-SARS-CoV-2 IgG antibody seroprevalence, Crete, July 2020

| **July** | **Positive/**  **sample size** | | **S1: Crude prevalence** | | **S2: Age, sex and population-adjusted prevalence** | | **S3: S2 + adjustment for sensitivity and specificity** | | **S4: S3 + NPHO data *** | |
| --- | --- | --- | --- | --- | --- | --- | --- | --- | --- | --- |
|  | **n/N** | | **Prevalence (%)** | **95% CI** | **Prevalence (%)** | **95% CI** | **Prevalence (%)** | **95% CI** | **Prevalence (%)** | **95% CI** |
| **Total** | 3/568 | | 0.53 | 0–1.12 | 0.37 | 0–0.88 | 0.09 | 0–0.69 | 0.09 | 0–0.69 |
| **Age group**  **(years)** | **0–29** | 0/173 | 0 | | 0 | | 0 | | 0 | |
|  | **30–49** | 1/183 | 0.55 | 0-1.61 | 0.41 | 0-1.33 | 0.13 | 0-1.23 | 0.13 | 0-1.23 |
|  | **50–69** | 1/129 | 0.78 | 0-2.29 | 0.92 | 0-2.56 | 0.74 | 0-2.70 | 0.74 | 0-2.70 |
|  | **≥ 70** | 1/83 | 1.20 | 0-3.55 | 0.43 | 0-1.83 | 0.15 | 0-1.83 | 0.15 | 0-1.83 |
| **Sex** | **Male** | 2/267 | 0.75 | 0-1.78 | 0.64 | 0–1.60 | 0.41 | 0–1.55 | 0.41 | 0–1.55 |
|  | **Female** | 1/301 | 0.33 | 0-0.98 | 0.11 | 0–0.50 | 0 | 0–0.24 | 0 | 0–0.24 |
| **‘Ν-1’ chi-squared test**  **Difference between sex** | | | Difference = 0.42%  p = 0.491 | | Difference = 0.53%  p = 0.292 | | Difference = 0.41%  p = 0.267 | | Difference = 0.41%  p = 0.267 | |
| **CFR (%)** | **95% CI** | | **IFR according to** | | | | | | | |
|  |  |  | **S1** | | **S2** | | **S3** | | **S4** | |
|  |  |  | **IFR (%)** | **95% CI** | **IFR (%)** | **95% CI** | **IFR (%)** | **95% CI** | **IFR (%)** | **95% CI** |
| 0 | | | 0 | | 0 | | 0 | | 0 | |

*NPHO: 9 cases, 0 deaths

**Table 3.** Anti-SARS-CoV-2 IgG antibody seroprevalence, Crete, August 2020

| **August** | | **Positive/**  **sample size** | **S1: Crude prevalence** | | **S2: Age, sex and population-adjusted prevalence** | | **S3: S2 + adjustment for sensitivity and specificity** | | **S4: S3 + NPHO data *** | |
| --- | --- | --- | --- | --- | --- | --- | --- | --- | --- | --- |
|  |  | **n/N** | **Prevalence (%)** | **95% CI** | **Prevalence (%)** | **95% CI** | **Prevalence (%)** | **95% CI** | **Prevalence (%)** | **95% CI** |
| **Total** | | 1/317 | 0.32 | 0-0.93 | 0.16 | 0–0.59 | 0 | 0–0.35 | 0.03 | 0–0.35 |
| **Age group**  **(years)** | **0–29** | 0/103 | 0 | | 0 | | 0 | | 0.03 | |
|  | **30–49** | 1/106 | 0.94 | 0-2.78 | 0.52 | 0-1.89 | 0.27 | 0-1.91 | 0.31 | 0-2.03 |
|  | **50–69** | 0/65 | 0 | | 0 | | 0 | | 0.03 | |
|  | **≥ 70** | 0/43 | 0 | | 0 | | 0 | | 0.02 | |
| **Sex** | **Male** | 0/150 | 0 | | 0 | | 0 | | 0.03 | |
|  | **Female** | 1/167 | 0.60 | 0- 1.77 | 0.31 | 0–1.15 | 0.01 | 0–1.02 | 0.04 | 0-1.06 |
| **‘Ν-1’ chi-squared test**  **Difference between sex** | | | **Difference = 0.60%**  **p = 0.343** | | **Difference = 0.31%**  **p = 0.496** | | **Difference = 0.01%**  **p = 0.903** | | **Difference = 0.01%**  **p = 0.962** | |
| **CFR (%)** | | **95% CI** | **IFR according to** | | | | | |  | |
|  |  |  | **S1** | | **S2** | | **S3** | | **S4** | |
|  |  |  | **IFR (%)** | **95% CI** | **IFR (%)** | **95% CI** | **IFR (%)** | **95% CI** | **IFR (%)** | **95% CI** |
| 0.49 | | (0-1.45) | 0.02 | (0.01-NA) | 0.03 | (0.01-NA) | 0.08 | (0.02-NA) | 0.08 | (0.02-NA) |

*NPHO: 204 cases, 1 death

**Table 4.** Anti-SARS-CoV-2 IgG antibody seroprevalence, Crete, September 2020

| **September** | | **Positive/**  **sample size** | **S1: Crude prevalence** | | **S2: Age, sex and population-adjusted prevalence** | | **S3: S2 + adjustment for sensitivity and specificity** | | | **S4: S3 + NPHO data *** | |
| --- | --- | --- | --- | --- | --- | --- | --- | --- | --- | --- | --- |
|  |  | **n/N** | **Prevalence (%)** | **95% CI** | **Prevalence (%)** | **95% CI** | **Prevalence (%)** | | **95% CI** | **Prevalence (%)** | **95% CI** |
| **Total** | | 4/554 | 0.72 | 0.02–1.43 | 1.30 | 0.36–2.25 | 1.20 | | 0.07–2.33 | 1.22 | 0.09-2.35 |
| **Age group**  **(years)** | **0–29** | 1/136 | 0.74 | 0-2.17 | 0.90 | 0-2.49 | 0.72 | | 0-2.62 | 0.73 | 0-2.63 |
|  | **30–49** | 2/183 | 1.09 | 0 -2.60 | 0.19 | 0-0.83 | 0 | | 0-0.63 | 0.03 | 0-0.66 |
|  | **50–69** | 1/148 | 0.68 | 0-2.00 | 4.36 | 1.07-7.65 | 4.85 | | 0.92-8.78 | 4.87 | 0.94-8.80 |
|  | **≥ 70** | 0/87 | 0 | | 0 | | 0 | | | 0 | |
| **Sex** | **Male** | 1/210 | 0.48 | 0-1.41 | 1.85 | 0.03–3.67 | 1.85 | | 0–4.03 | 1.87 | 0-4.05 |
|  | **Female** | 3/344 | 0.87 | 0-1.85 | 0.75 | 0–1.65 | 0.53 | | 0–1.62 | 0.55 | 0-1.64 |
| **‘Ν-1’ chi-squared test**  **Difference between sex** | | | Difference = 0.39%  p = 0.599 | | Difference = 1.10%  p = 0.243 | | Difference = 1.32%  p = 0.136 | | | Difference = 1.32%  p = 0.140 | |
| **CFR (%)** | | **95% CI** | **IFR according to** | | | | | | | | |
|  |  |  | **S1** | | **S2** | | **S3** | | | **S4** | |
|  |  |  | **IFR (%)** | **95% CI** | **IFR (%)** | **95% CI** | **IFR (%)** | **95% CI** | | **IFR (%)** | **95% CI** |
| 0.84 | | (0-2.48) | 0.01 | (0.01-0.80) | 0.01 | (0.005-0.04) | 0.01 | (0.006-0.23) | | 0.01 | (0.006-0.18) |

*NPHO: 116 cases, 1 death

**Table 5.** Anti-SARS-CoV-2 IgG antibody seroprevalence, Crete, October 2020

| **October** | | **Positive/**  **sample size** | **S1: Crude prevalence** | | **S2: Age, sex and population-adjusted prevalence** | | **S3: S2 + adjustment for sensitivity and specificity** | | **S4: S3 + NPHO data *** | |
| --- | --- | --- | --- | --- | --- | --- | --- | --- | --- | --- |
|  |  | **n/N** | **Prevalence (%)** | **95% CI** | **Prevalence (%)** | **95% CI** | **Prevalence (%)** | **95% CI** | **Prevalence (%)** | **95% CI** |
| **Total** | | 3/312 | 0.96 | 0–2.04 | 1.44 | 0.12–2.77 | 1.37 | 0–2.95 | 1.41 | 0.04-3.01 |
| **Age group**  **(years)** | **0–29** | 1/82 | 1.22 | 0-3.60 | 2.47 | 0-5.83 | 2.59 | 0-6.60 | 2.63 | 0.04-6.64 |
|  | **30–49** | 2/104 | 1.92 | 0 -4.56 | 1.90 | 0-4.53 | 1.92 | 0-5.05 | 1.97 | 0.05-5.10 |
|  | **50–69** | 0/76 | 0 | | 0 | | 0 | | 0.04 | |
|  | **≥ 70** | 0/50 | 0 | | 0 | | 0 | | 0.02 | |
| **Sex** | **Male** | 2/135 | 1.48 | 0-3.65 | 2.28 | 0.4.80 | 2.37 | 0–5.38 | 2.41 | 0.04–5.42 |
|  | **Female** | 1/177 | 0.56 | 0-1.67 | 0.62 | 0–1.77 | 0.38 | 0–1.76 | 0.42 | 0–1.80 |
| **‘Ν-1’ chi-squared test**  **Difference between sex** | | | Difference = 0.98%  p = 0.391 | | Difference = 3.44%  p = 0.0.38 | | Difference = 1.99%  p = 0.014 | | Difference = 4.11%  p = 0.015 | |
| **CFR (%)** | | **95% CI** | **IFR according to** | | | | | | | |
|  |  |  | **S1** | | **S2** | | **S3** | | **S4** | |
|  |  |  | **IFR (%)** | **95% CI** | **IFR (%)** | **95% CI** | **IFR (%)** | **95% CI** | **IFR (%)** | **95% CI** |
| 1.29 | | (0-2.74) | 0.03 | (0.01-NA) | 0.02 | (0.01-0.20) | 0.02 | (0.01-NA) | 0.02 | (0.01-0.68) |

*NPHO: 242 cases, 3 deaths

**Table 6.** Anti-SARS-CoV-2 IgG antibody seroprevalence, Crete, November 2020

| **November** | | **Positive/**  **sample size** | **S1: Crude prevalence** | | **S2: Age, sex and population-adjusted prevalence** | | **S3: S2 + adjustment for sensitivity and specificity** | | **S4: S3 + NPHO data *** | |
| --- | --- | --- | --- | --- | --- | --- | --- | --- | --- | --- |
|  |  | **n/N** | **Prevalence (%)** | **95% CI** | **Prevalence (%)** | **95% CI** | **Prevalence (%)** | **95% CI** | **Prevalence (%)** | **95% CI** |
| **Total** | | 8/424 | 1.89 | 0.59–3.18 | 1.35 | 0.25–2.45 | 1.26 | 0–2.57 | 1.45 | 0.19-2.76 |
| **Age group**  **(years)** | **0–29** | 3/110 | 2.73 | 0-5.77 | 1.19 | 0-3.22 | 1.07 | 0-3.49 | 1.24 | 0.17-3.66 |
|  | **30–49** | 4/154 | 2.60 | 0.09 -5.11 | 2.91 | 0.26-5.56 | 3.12 | 0-6.29 | 3.32 | 0.20-6.49 |
|  | **50–69** | 0/103 | 0 | | 0 | | 0 | | 0.22 | |
|  | **≥ 70** | 1/57 | 1.75 | 0-5.16 | 0.44 | 0-2.17 | 0.17 | 0-2.23 | 0.31 | 0.14-2.37 |
| **Sex** | **Male** | 2/156 | 1.28 | 0-3.05 | 1.65 | 0-3.64 | 1.61 | 0-3.99 | 1.80 | 0.19-4.18 |
|  | **Female** | 6/268 | 2.24 | 0.47-4.01 | 1.06 | 0–2.29 | 0.91 | 0–2.38 | 1.10 | 0.19–2.57 |
| **‘Ν-1’ chi-squared test**  **Difference between sex** | | | Difference = 0.96%  p = 0.484 | | Difference = 0.59%  p = 0.602 | | Difference = 0.70%  p = 0.518 | | Difference = 0.70%  p = 0.549 | |
| **CFR (%)** | | **95% CI** | **IFR according to** | | | | | | | |
|  |  |  | **S1** | | **S2** | | **S3** | | **S4** | |
|  |  |  | **IFR (%)** | **95% CI** | **IFR (%)** | **95% CI** | **IFR (%)** | **95% CI** | **IFR (%)** | **95% CI** |
| 1.37 | | (0.70-2.04) | 0.09 | (0.05-0.43) | 0.10 | (0.06-0.64) | 0.11 | (0.06-NA) | 0.10 | (0.06-1.11) |

*NPHO: 1167 cases, 16 deaths

**Table 7.** Anti-SARS-CoV-2 IgG antibody seroprevalence, Crete, December 2020

| **December** | | **Positive/**  **sample size** | **S1: Crude prevalence** | | **S2: Age, sex and population-adjusted prevalence** | | **S3: S2 + adjustment for sensitivity and specificity** | | **S4: S3 + NPHO data *** | |
| --- | --- | --- | --- | --- | --- | --- | --- | --- | --- | --- |
|  |  | **n/N** | **Prevalence (%)** | **95% CI** | **Prevalence (%)** | **95% CI** | **Prevalence (%)** | **95% CI** | **Prevalence (%)** | **95% CI** |
| **Total** | | 18/747 | 2.41 | 1.31-3.51 | 2.42 | 1.42-3.41 | 2.53 | 1.34-3.72 | 2.58 | 1.38-3.77 |
| **Age group**  **(years)** | **0–29** | 5/246 | 2.03 | 0.27-3.80 | 2.44 | 0.74-4.15 | 2.56 | 0.53-4.59 | 2.59 | 0.55-4.62 |
|  | **30–49** | 7/221 | 3.17 | 0.86 -5.48 | 3.28 | 1.19-5.38 | 3.57 | 1.06-6.07 | 3.62 | 1.11-6.12 |
|  | **50–69** | 4/157 | 2.55 | 0.08-5.01 | 2.02 | 0-4.27 | 2.05 | 0-4.74 | 2.13 | 0.08-4.82 |
|  | **≥ 70** | 2/123 | 1.63 | 0-3.86 | 1.04 | 0-2.91 | 0.88 | 0-3.12 | 0.94 | 0.06-3.18 |
| **Sex** | **Male** | 8/365 | 2.19 | 0.69-3.69 | 1.80 | 0.49-3.10 | 1.79 | 0.23-3.35 | 1.85 | 0.29-3.41 |
|  | **Female** | 10/382 | 2.62 | 1.02-4.22 | 3.02 | 1.50-4.53 | 3.25 | 1.44-5.06 | 3.30 | 1.49-5.11 |
| **‘Ν-1’ chi-squared test**  **Difference between sex** | | | Difference = 0.43%  p = 0.702 | | Difference = 1.22%  p = 0.279 | | Difference = 1.46%  p = 0.205 | | Difference = 1.45%  p = 0.213 | |
| **CFR (%)** | | **95% CI** | **IFR according to** | | | | | | | |
|  |  |  | **S1** | | **S2** | | **S3** | | **S4** | |
|  |  |  | **IFR (%)** | **95% CI** | **IFR (%)** | **95% CI** | **IFR (%)** | **95% CI** | **IFR (%)** | **95% CI** |
| 7.77 | | (1.52-4.78) | 0.09 | (0.06-0.25) | 0.09 | (0.06-0.23) | 0.10 | (0.06-0.28) | 0.09 | (0.06-0.26) |

*NPHO: 309 cases, 24 deaths
